# Data on insulin therapy refusal among type II diabetes mellitus patients in Mashhad, Iran

**DOI:** 10.1016/j.dib.2018.04.136

**Published:** 2018-05-04

**Authors:** Zahra Mostafavian, Sahar Ghareh, Farnaz Torabian, Mohammad Sarafraz Yazdi, Mahmood Reza Khazaei

**Affiliations:** a Department of Community Medicine, Mashhad Branch, Islamic Azad University, Mashhad, Iran; b Department of Internal Medicine, Mashhad Branch, Islamic Azad University, Mashhad, Iran; c Medical Student, Faculty of Medicine, Islamic Azad University, Mashhad Branch, Mashhad, Iran; d Department of Internal Medicine, Mashhad Branch, Islamic Azad University, Mashhad, Iran; e Department of Pediatric Medicine, Mashhad Branch, Islamic Azad University, Mashhad, Iran

**Keywords:** Insulin therapy, Refusal, Type 2 diabetes mellitus, Iran

## Abstract

Insulin has been considered as a therapy option of last resort in type 2 diabetes (T2DM) management. Delay in insulin therapy is common in these patients. This study collected the data on the factors associated with insulin refusal in poorly controlled T2DM patients prior to insulin therapy. The data collected from two endocrinology outpatient clinics affiliated by Islamic Azad University of Mashhad, Iran (IAUM) from January 2016 to September 2017. Study population was adults with non-insulin-using type 2 diabetes mellitus who refused insulin therapy. A 17-items researcher made questionnaire was used to obtain demographic data and information toward causes of insulin refusal. Data were analyzed using SPPS V.16 with descriptive and analytical tests such as multiple logistic regressions. The data of 110 patients with T2DM was recorded in this study. The most prevalent cause of insulin therapy refusal was reported to be painful insulin injection (78.2%) followed by this item “I’m afraid of injecting myself with a needle” (74.5%). Regression analysis revealed that education level had a significant association with the item of “Injecting insulin is painful” (P=0.033, OR=0.357). Also age (P=0.025, OR=1.076) and disease duration (P=0.024, OR=0.231) were significantly associated with the question “taking insulin makes life less flexible”. Several causes have been found regarding misconceptions about insulin therapy in T2DM patients. Specialized educational interventions are recommended for initiating successful insulin therapy in these patients.

**Specifications Table**

**Table:** 

Subject area	Medicine
More specific subject area	Diabetes, Endocrinology and Metabolism
Type of data	Tables
How data was acquired	The data acquired from two endocrinology outpatient clinics affiliated by Islamic Azad University of Mashhad, Iran (IAUM) from January 2016 to September 2017. Study population was adults with non-insulin-using type 2 diabetes mellitus who refused insulin therapy. A 17-items researcher made questionnaire was used to obtain demographic data and information toward causes of insulin refusal.
Data format	Raw, analyzed
Experimental factors	The questionnaire consisted of 17 two-way questions. In the test-retest method, the questionnaire was completed by 15 patients and again completed 2 weeks later by the same patients.
Experimental features	Multiple logistic regressions were used to study the association between cause of insulin therapy refusal and some variables including age, gender, marital status, education level, and disease duration.
Data source location	Mashhad, Razavi Khorasan province, Iran
Data accessibility	Data are included in this article

**Value of the data**

• Many type 2 diabetes mellitus (T2DM) patients are reluctant to start insulin therapy and may delay beginning it for significant periods of time [1]. Causes of delay are multi-factorial, such as healthcare system barriers and clinical attitudes among healthcare professionals. Delay may also be due to patients experiencing PIR [2].
• Refusal or delay for insulin therapy may induce many adverse effects on patients.
• While other studies explored patients’ attitudes toward insulin therapy, limited data are available regarding patients׳ attitudes prior to treatment.
• The data presented in this article shows the factor affecting insulin refusal in poorly controlled T2DM patients prior to insulin therapy.
• The data in this article indicates that pain and fear from injection are the most important factors for insulin refusal.
• The cost of insulin was recognized as one of the most important factors for insulin refusal.

## 1 Data

A total of 110 eligible patients were enrolled in this study. The response rate in this study was 100%. Patients’ mean age was 56.61 ± 12.19 (33–88) years. Furthermore, 77.3% of patients were female and 73.6% of patients had positive family history for diabetes. The majority of the respondents were married (95.5%), and had only primary education or no formal education (68.2%). 82.7% of the patients had been diagnosed with T2DM for more than ten years. Demographic data are listed in Table 1.

**Table 1:** Patients׳ demographic data.

**Variables**		**Frequency**	
		**Number**	**Percent**
Gender	Female	85	77.3
	Male	25	22.7
Marital Status	Single	5	4.5
	Married	105	95.5
Education Level	Less than Diploma	75	68.2
	Diploma	21	19.1
	Formal Education	14	12.7
Medication	Metformin	22	20
	Metformin plus other agents	82	80
Diabetes Family History	Positive	81	73.6
	Negative	29	23.4
Diabetes Diagnosis Duration	Ten years and more	91	82.7
	Less than ten years	19	17.3
Total	–	110	100

The causes of insulin refusal according to patients׳ opinion are listed in Table 2. As seen in Table 2, the most prevalent cause of insulin therapy refusal was reported painful insulin injection (78.2%). Second more prevalent reported cause was “I’m afraid of injecting myself with a needle” (74.5%). In our study, 34.5% of patients expressed the cost of insulin therapy as a reason for refusing it.

**Table 2:** Causes of insulin refusal according to patients׳ opinion.

**Parameters associated with insulin therapy refusal**	**Number (%)**	
	**Agree**	**Disagree**
Taking insulin means I have failed to manage my diabetes with diet and tablets	16 (14.5%)	94 (85.5%)
Taking insulin means other people look at me as a sick person	8 (7.3%)	102 (92.7%)
Taking insulin makes it more difficult to fulfill my responsibilities (at work, at home)	6 (5.5%)	104 (94.5%)
Injecting insulin is painful	86 (78.2%)	24 (21.8%)
Taking insulin is embarrassing	10 (9.1%)	100 (90.9%)
Insulin is expensive	38 (34.5%)	72 (65.5%)
It is difficult to inject the right amount of insulin correctly at the right time every day	18 (16.4%)	92 (83.6%)
Managing insulin injections takes a lot of time	6 (5.5%)	104 (94.5%)
Taking insulin means I have to give up activities I enjoy	33 (30%)	77 (70%)
Taking insulin means my health will deteriorate	19 (17.3%)	91 (82.7%)
Taking insulin decreases my self confidence	13 (11.8%)	97(88.2%)
Taking insulin makes me more dependent on my doctor	20 (18.2%)	90 (81.8%)
Insulin causes weight gain	8 (7.3%)	102 (92.7%)
Being on insulin causes family and friends to be more concerned about me	20 (18.2%)	90 (81.8%)
Taking insulin increases the risk of low blood glucose levels (hypoglycaemia)	24 (21.8%)	86 (78.2%)
I’m afraid of injecting myself with a needle	82 (74.5%)	28 (25.5%)
Taking insulin causes renal failure and blindness	13 (11.8%)	97 (88.2%)

We found that education level had a statistically significant association with fifth item of questionnaire “painful insulin injection” among patients (*P*=0.033, OR=0.357). In this way, having less education was correlated to this item. Patients age (*P*=0.025, OR=1.076) and disease duration (*P*=0.024, OR=0.231) were significantly associated with the eleventh question of questionnaire i.e. “taking insulin makes life less flexible”. That means, older patients, as well as individuals with a disease duration of more than or equal to one year, more agree with the eleventh item of questionnaire i.e. (insulin therapy reduces the flexibility of life). There was no significant relationship between demographic variables and other 15 questions of the questionnaire.

## 2 Experimental design, materials and methods

This cross-sectional study was carried using 110 patients with diabetes type 2 who referred to two endocrinology outpatient clinic affiliated to Islamic Azad University of Mashhad, Iran (IAUM) from January 2016 to September 2017. Convenience sampling was done in the study. They were selected while attending to clinic for follow-up of their disease. All participants gave oral consent before data gathering which was done by one clinician. Inclusion criteria were: T2DM patients had not received insulin, age≥18 years, poor glycemic control, HbA1c >7.0%, oral glucose-lowering therapy, and patients who refused insulin therapy. Patients were excluded if they had T1DM, severe psychiatric illness, dementia, unstable cardiovascular disease, an existing debilitating medical condition, unwilling to give consent, and were on current insulin treatment.

Our tool for data gathering was a self-administered questionnaire developed by a group of endocrinologists and Community medicine specialists according to some studies agents [2], [3]. The questionnaire had two parts. In part one, general information including demographic and clinical data, such as age, gender, marital status, education level, drug history, family history of diabetes mellitus, duration of diabetes, fasting blood glucose (FBS) and HbA1c levels were obtained from the patients. In the second part, the questionnaire consisted of 17 items. We examined causes of unwillingness to use insulin. The answer to each question is in the form of two options, including agree and disagree. Face and content validity was confirmed by the experts. The Cronbach׳s alpha value for the questionnaire was 0.72. Also, in the test-retest method, the questionnaire was completed by 15 patients and again completed 2 weeks later by the same patients. For each question, the intra-class correlation coefficient was calculated and was higher than 0.9 for all of the items.

Statistical analyses were performed by SPSS version 16.0 software using descriptive and analytical tests. Multiple logistic regressions were used to study the association between cause of insulin therapy refusal and some variables including age, gender, marital status, education level, and disease duration. Reference category for variables was as follows; gender: female sex, educational status: less than diploma, marital status: single and for the duration of the illness: more than or equal to one year. Statistical significance was set at *P*<0.05.

This study was reviewed and approved by the Medical Research Ethics Committee of IAUM. (Ethical Code: IR.IAU.MSHD.REC.1396.38).
